# MicroRNA-29a-3p enhances dental implant osseointegration of hyperlipidemic rats via suppressing dishevelled 2 and frizzled 4

**DOI:** 10.1186/s13578-018-0254-y

**Published:** 2018-10-26

**Authors:** Fei Liu, Zhifeng Wang, Fangfang Liu, Jinzhao Xu, Qibo Liu, Kaifeng Yin, Jing Lan

**Affiliations:** 1 Department of Prosthodontics, School of Stomatology, Shandong University, Jinan, 250000 China; 2 Shandong Provincial Key Laboratory of Oral Tissue Regeneration, 44-1 West Wenhua Street, Jinan, 250012 Shandong China; 3 Department of Pediatric Dentistry, School of Stomatology, Shandong University, Jinan, 250000 China; 4 Department of Orthodontics, Herman Ostrow School of Dentistry, Los Angeles, CA 90089 USA; 5 Center for Craniofacial Molecular Biology, Herman Ostrow School of Dentistry, Los Angeles, 90033 USA; 6 Department of Implantology, Stomatological Hospital of Nanyang, Nanyang, 473000 China; 7 Department of Prosthodontics, School of Dentistry, Shandong University, Jinan, China

**Keywords:** miR-29a-3p, Hyperlipidemia, Dvl2, Fzd4, Osseointegration

## Abstract

**Background:**

Fine osseointegration is the basis of long-term survival of implant. In our previous study, we observed a strong correlation between hyperlipidemia and compromised osseointegration. MicroRNA-29a-3p (miR-29a-3p) has been discovered to participate in bone marrow mesenchymal stem cells (BMSCs) differentiation. However, the role and the underlying mechanisms of hyperlipidemia and miR-29a-3p in osseointegration still remain obscure.

**Results:**

In peri-implant bone tissues of hyperlipidemia rats, bone mass, mineralization and bone trabecula formation were weakened. Alkaline phosphatase (ALP) and runt-related transcription factor 2 (Runx2), and miR-29a-3p expression were reduced. While in normal rats, implant-bone interfaces were filled with dense new bone and ALP, Runx2 and miR-29a-3p were up-regulated. Overexpressed miR-29a-3p can reverse the adverse effect of hyperlipidemia on osseointegration. Implants were tightly integrated with the surrounding dense new bone tissues, and ALP as well as Runx2 mRNAs were enhanced in miR-29a-3p overexpressed and hyperlipidemia rats, while little peri-implant bone tissue existed, ALP and Runx2 deregulated on miR-29a-3p inhibited rats. Dishevelled 2 (Dvl2) mRNA was declined in peri-implant bone tissue of high-fat (HF) group than normal group, while frizzled 4 (Fzd4) mRNA declined on day 5 and increased from day 10 to day 20 after implantation in hyperlipidemia rats than in normal rats. Next, BMSCs were cultured under HF or normal medium in vitro. In the HF group, ALP activity and mineralization, ALP and Runx2 mRNAs and proteins expression, and miR-29a-3p expression were suppressed, while adipogenesis was increased, as a result, cytoskeletons were sparse and disordered compared to control group. However, when miR-29a-3p was overexpressed in BMSCs, ALP activity, ALP, Runx2, Dvl2 and Fzd4 mRNAs and proteins expressions were up-regulated. As miR-29a-3p was inhibited in BMSCs, the reverse results were obtained. In addition, promoter assay revealed that miR-29a-3p can directly suppress Wnt/β-catenin pathway related Dvl2 and Fzd4 through binding to their 3′-UTR.

**Conclusions:**

MiR-29a-3p facilitated implant osseointegration via targeting Wnt/β-catenin pathway-related Dvl2 and Fzd4. MiR-29a-3p/Dvl2/Fzd4 may serve as a promising therapeutic target for hyperlipidemia osseointegration.

## Background

High-fat diet (HFD) can induce hyperlipidemia, which was characterized by an elevation of lipids in the bloodstream. Hyperlipidemia significantly damaged bone metabolism, decreased bone mineral density, volume and strength, and increased the risk of bone fracture and osteoporosis [1–3]. Excellent osseointegration was associated with long-term success rate of implants. However, hyperlipidemia leaded to increased implant loss, and reduced strength and bone formation of implant-bone interface [3–5].

As the incidence of hyperlipidemia has been increasing yearly [6], it is significant to study the underlying molecular mechanism of hyperlipidemia on implant osseointegration. Bone marrow mesenchymal stem cells (BMSCs), known for its pluripotent differentiation capacity, were the progenitor cells for osteoblasts, and played a substantial role in bone formation and regeneration [7, 8]. Xu et al. [9] demonstrated that BMSCs transplantation significantly enhanced new bone formation, but hyperlipidemia compromised homing efficiency of systematically transplanted BMSCs and inhibited bone regeneration. Alkaline phosphatase (ALP), engaged in bone matrix mineralization, and runt-related transcription factor 2 (Runx2), a master transcription factor of osteogenesis, were considered as early marker genes of osteogenesis [10–17].

A great amount of microRNAs (miRNAs) has been shown to regulate bone formation and regeneration as well as implant osseointegration [18, 19]. Many miRNAs were aberrantly expressed during osteogenesis in hyperlipidemia models, including miR-29b, miR-17, miR-146, miRNA-23, miR-34c. [20–24]. Interestingly, miR-29a-3p has been confirmed as a positive regulator on osteogenesis [25–27], and was increased during osteogenic differentiation in mice [27], but its role in osseointegration is not clear.

Wnt/β-catenin pathway played significant roles in osteogenic differentiation of BMSC [28–30]. Osteogenesis was enhanced by the activation of Wnt/β-catenin pathway [31]. Accordingly, it is important to search for Wnt/β-catenin signaling-related downstream genes of miR-29a-3p to understand the underlying mechanism of miR-29a-3p on osseointegration.

Based on previous studies [32, 33], we have successfully established hyperlipidemia rat models by inserting implants into the bilateral femoral metaphysis, a method widely adopted by other researches [33–35]. To further elucidate the detailed function of miR-29a-3p during BMSCs osteogenesis and the effect of hyperlipidemia on miR-29a-3p, relevant biological and functional assays were performed in vitro. Furthermore, online softwares were used to screen the Wnt/β-catenin pathway mediated downstream target genes of miR-29a-3p. Dishevelled 2 (Dvl2), a cytoplasmic molecule of the Wnt/β-catenin signaling [36, 37], and frizzled 4 (FZD4), an essential receptor of the Wnt/β-catenin signal pathway [38, 39], were selected and validated as downstream targets of miR-29a-3p by dual luciferase reporter assays.

## Methods

### Biological and functional experiments in vivo

#### Animal preparation

All Wistar rats (male, 6 weeks old and 180–220 g weigh), were purchased from the Experimental Animal Center of Shandong University (Ji’nan, China). All rats were housed in sterilized, pathogen-free, temperature controlled facility on a normal 12-h light/dark cycle, and standard diet and water were provided ad libitum.

Rats were randomly divided into normal and HF group (n = 16 in each group). HF group was fed on HFD (78.8% basic feed, 10% lard, 10% egg yolk powder, 1% cholesterol, 0.2% bile salt), while normal group fed on normal diet. After 8 weeks, fasting blood samples were taken from the endocanthion vein of animals to determine serum lipid levels including low density lipoprotein (LDL), high density lipoprotein (HDL), triglyceride (TG) and total cholesterol (TC) using an autoanalyzer (Hitachi, Japan).

#### Implant placement and tissue preparation

General anesthesia was obtained by intraperitoneal injection of 10% chloral hydrate (10 ml/kg, Qilu Hospital, Jinan, China). For implant insertion, a hole (1.3 mm diameter) was drilled on the site 5 mm away from the bilateral distal femoral metaphysis. Subsequently, the titanium implant (Shuangyang, China), rodshaped custom-made, 1.5 mm diameter and 2.5 mm length was inserted into the hole [33–35]. After operation, all rats received intramuscularly injection of penicillin (0.06 ml/kg, once a day) for 3 postoperative days. 5, 10, 15, 20 days after implantation, 4 rats in each group were sacrificed by deep anesthesia with chloral hydrate. 1 mm bones around implants were dissected without surrounding muscles. All samples were frozen in liquid nitrogen rapidly.

#### Hard tissue slices preparation and hematoxylin–eosin (HE) staining

The samples, 10 days after implantation, were prepared for hard tissue slices. After fixation with 4% paraformaldehyde, the bone tissue with implant were embedded with resin, and sectioned in longitudinal direction parallel to the long axis of the implant by section cutter. About 30 μm thick sections were obtained by grinding machine (E400CS, EXAKT Vertriebs Gmbh, Germany) and stained with hematoxylin–eosin (HE) by staining parallel adhesive tablet device (E401/402, EXAKT Vertriebs Gmbh, Germany). Implant-bone interface were observed by microscopy (Olympus, Japan).

#### RNA extraction and quantitative reverse-transcription polymerase chain reaction(qRT-PCR)

5, 10, 15, 20 days after insertion, 1 mm femur tissues around implants were prepared for qRT-PCR. Total RNA of these bone tissues was isolated using Trizol reagent (TaKaRa, China) according to the manufacture’s instruction. The first-strand cDNA was synthesized from 1 μg of total RNA using PrimeScript™ II 1st Strand cDNA Synthesis Kit (Takara, Dalian, China). Quantitative reverse-transcription polymerase chain reaction (qRT-PCR) was conducted with a standard SYBR Green PCR kit (TaKaRa, China) on the LightCycler 480II96 (Roche, Switzerland). The relative expressions of miR-29a-3p, and mRNA level expression of ALP, Runx2 were measured using the 2^−ΔΔCt^ methods with GAPDH as an internal control. Primer sequences are listed in Table 1.

**Table 1:** Primers used for qRT-PCR

Gene	Forward primer sequence (5′–3′)	Reverse primer sequence (5′–3′)
ALP	TGAGCGACACGGACAAGAAG	GCCTGGTAGTTGTTGTGAGCAT
Runx2	CACAAGTGCGGTGCAAACTT	AATGACTCGGTTGGTCTCGG
Dvl2	TCCACCATTACCCCCTTTGC	GCCATGCTCACTGCTGTCT
Fzd4	GGAAGGACCAGGTGACGAAG	GGAATATGATGGGGCGCTCA
GAPDH	TGATGGGTGTGAACCACGAG	CTGATGGGTGTGAACCACGAG
miR-29a-3p	UAACCGAUUUCAAAUGGUGCUA	

#### Lentiviral vectors construction, miR-29a-3p function assay

Recombinant lentiviral expression vectors were recruited to establish lenti-miR-29a-3p overexpressed vector (miR-29a-3p-enhancer), lenti-miR-29a-3p deregulated vector (miR-29a-3p-inhibitor) and negative control vectors (enhancer-nc and inhibitor-nc) (RiboBio, Guangzhou, China).

Male Wistar rats, fed with HFD for 8 weeks, were used in this experiment. These recombinant lentiviral expression vectors were intramuscularly injected into the femoral metaphysis region (n = 8 in each group). 3 days after injection, implants were inserted consistent with the above method. 10 days after implantation, all animals were sacrificed and 1 mm bone tissues around implants were obtained. HE stained hard tissue slices and qRT-PCR methods were performed according to the previously described protocols.

### Biological and functional assays in vitro

#### BMSCs culture, identification and induction

BMSCs were isolated from male Wistar rat (3 weeks old) and cultured as described previously [40]. BMSCs (1 × 106) at passage 3 were incubated with antibodies against mouse CD11, CD11b, CD44, CD45, CD90 (BioLegend, USA) and CD29 (eBioscience, USA), fluorescence-Activated Cell Sorting (FACS) was performed by using CXP Analysis 2.1 software (Beckman Coulter, USA). BMSCs of passage 3 were randomly divided into control and experiment groups, control group was induced in osteogenic medium (10% FBS α-MEM culture medium, 50l g/ml ascorbic acid, 10 nM dexamethasone, and 10 mM b-glycerophosphatase) and experiment group cultured in HF osteogenic medium (Xingzhi Technology, Guangzhou, China).

#### Immunofluorescence analysis

Immunofluorescence analysis was performed to observe the location of ALP and Runx2 proteins and cytoskeletal structures of BMSCs. BMSCs were cultured and fixed in a 6-well culture board and incubated with antibodies specific for ALP (1:1000, Abcam, USA) or Runx2 (1:1000, Abcam, USA). After incubating with goat anti-mouse IgG (1:1000, Abcam, USA), the cystoskeleton was stained by phalloidin (1:200, Solarbio, China) and the nuclei was stained by adding DAPI (1:1000, Invitrogen, D3571). Finally the cells were observed with confocal laser scanning microscope (CLSM, LSM 780, CalZeissAG, Germany), nuclei were counted conducted by ZEISS ZEN SYSTEM 2012 software.

#### Alkaline phosphatase (ALP) staining and ALP activity assays

ALP staining and ALP activity assays were used to analyze ALP activity of BMSCs. 7, 14 days after osteogenic induction, BMSCs were washed with PBS and fixed in 4% paraformaldehyde for 20 min, and stained by BCIP/NBT ALP Color Development Kit (Beyotime, Shanghai, China) for 15 min in the dark. After thorough washing with PBS, images were taken using a microscope (Olympus, Japan). An ALP Colorimetric Assay Kit (Jiancheng Biotechnology, Nanjing, China) was used to quantify the ALP expression in control and experiment groups. The data were normalized to the corresponding total protein contents, which were determined using Enhanced BCA Protein Assay Kit (Beyotime Institute of Biotechnology, China).

#### Alizarin red S (ARS) staining

BMSCs of control and experiment groups were induced for 28 days, while BMSCs of non-induced group received no induction. ARS staining was conducted to assess the mineralization of BMSCs. The cells were fixed in 2% formaldehyde solution, washed with ddH_2_O and then stained for Ca deposit using 0.5% ARS (pH 4.0) (Sigma, Germany) for 20 min. Then inverted microscope was used to observe.

#### Oil red O staining

To assess the adipogenesis capability of BMSCs, cells (14 days after induction or non-induced) were stained with Oil Red O (Sigma, China) at 37 °C for 30 min, rinsed with 75% alcohol until the intercellular substance was clear. Cells were then observed and photographed under an inverted microscope.

#### RT-PCR

3, 5, 7, 14 days after induction, cells of control and experiment groups were collected. qRT-PCR was conducted to analyze ALP, Runx2 mRNA and miR29a-3p expressions,and the methods were consistent with the above.

#### Western blotting

Cells of these two groups were gathered on day 3, 5, 7, 14 after induction. Total proteins were extracted and quantified by the BCA Protein Quantitation Kit (Beyotime Biotechnology, China). 0.05ug of protein from each sample were loaded on SDS-PAGE gel and then transferred to PVDF membranes (Millipore, Billerica, MA, USA). Transferred membrane was immunoblotted with primary antibodies (Table 2). After incubation with secondary antibody (goat anti-rabbit IgG, Abcam, USA), protein levels were detected using the Western-Light chemiluminescent detection system (Peiqing, Shanghai, China).

**Table 2:** Antibodies used for western blotting

Name	Description	Manufacturer
Anti-ALP	Rabbit monoclonal, 140 kDa	CST (#3192)
Anti-Runx2	Rabbit monoclonal, 170 kDa	CST (#3179)
Anti-Dvl2	Mouse monoclonal, 65 kDa	Abcam (ab181770)
Anti-Fzd4	Rabbit monoclonal, 70 kDa	CSB-PA706537

#### Lenti-vectors transfection, miR-29a-3p function analysis in vitro

BMSCs were transfected with different concentrations of FAM-siRNA when cells reached 30–50% confluency. 24, 48 and 72 h after incubation, we analyzed the absorbance at 450 nm to detect the optimal concentration. MiR-29a-3p-enhancer, miR-29a-3p-inhibitor and enhancer/inhibitor-nc (RiboBio, Guangzhou, China) were transfected into BMSCs respectively. After 48 h, ALP staining was performed to demonstrate ALP activities of these four groups. RT-PCR and Western blotting were used to detect the mRNA and protein levels expressions of miR-29a-3p, ALP, Runx2, Fzd4 and Dvl2.

#### Luciferase reporter assay

Luciferase reporter assay was performed to detect whether miR-29a-3p targetly regulate Dvl2 and Fzd4. Luciferase reporters were generated based on the pMIR-REPORT vector (Sangon Biotech, Shanghai, China). The 3′-UTR sequence of Dvl2/Fzd4 and the mutant of these sequences, containing the predicted miR-29a-3p binding site, were cloned into the pMIR-REPORT vector, respectively. The luciferase reporter was co-transfected with miR-29a-3p enhancer, miR-29a-3p-mut enhancer, or miR-negative control (miR-NC) into 293T cells by Lipofectamine 3000 according to the manufacturer’s guidelines. The relative luciferase activity was measured with the Dual-Luciferase Reporter Assay System Kit (Beyotime, China). The experiments were performed independently in triplicate.

### Statistical analysis

All the data were analyzed by Graphpad Prism 7.0 software (GraphPad Software Inc., La Jolla, CA, USA). The mean differences between the two groups were compared by t-test, and the mean differences among three or more groups were compared by analyzed by one-way ANOVA. P < 0.05 from a two-tailed test was considered significant.

## Result

### Hyperlipidemia hindered osseointegration and reduced miR-29a-3p expression

**Fig. 1:**
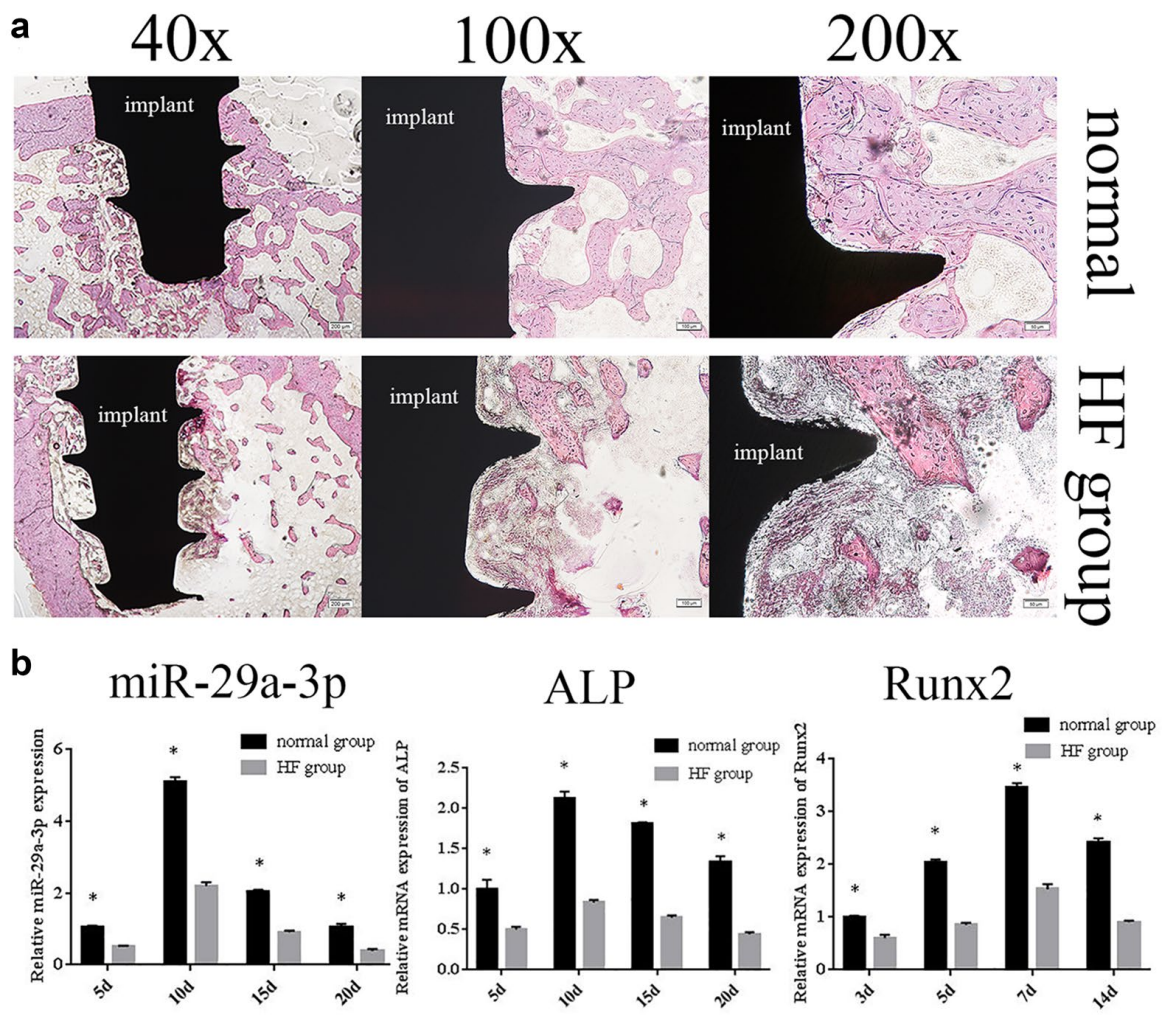
MiR-29a-3p expression was deregulated in hyperlipidemia rats along with impaired osseointegration. **a** HE stained hard tissue slices were used to assess the osseointegration of implant periphery bone both of normal and HF groups. **b** qRT-PCR analysis was used to determine the relative expression levels of miR-29a-3p, ALP mRNA and Runx2 mRNA in peri-implant bone tissues of HF group and normal group. Results were represented as mean ± SD. *P < 0.05

To determine the roles of hyperlipidemia in osseointegration and miR-29a-3p expression in vivo, hyperlipidemia animal models were established by HFD. Serum lipid levels of the animals from the two groups are listed in Table 3. TC increased by about twofolds, and TG and LDL obtained significantly increase in the HF group comparing to the normal group (*P *< 0.05), this result confirmed that our model successfully resembled hyperlipidemia in HF groups. HE images showed the bone mass, bone density and new bone volume were significantly reduced, and bone tissue was sparse on HF group, while implants were tightly integrated with the surrounding bone tissues on normal group. Moreover, many lightly stained osteoblasts were visible under high magnification in the normal group (Fig. 1a).

**Table 3:** The serum TC, TG, HDL and LDL of two groups

	TC	TG	HDL	LDL
Normal range	1.64 ± 0.21	1.01 ± 0.46	0.91 ± 0.15	0.24 ± 0.05
Control group	1.45 ± 0.22	0.73 ± 0.12	1.10 ± 0.16	0.29 ± 0.66
Experiment group	3.45 ± 0.65*	1.74 ± 0.68*	1.07 ± 0.22*	0.65 ± 0.12*

Values are mean ± SD in mmol/l, *P < 0.05 was considered statistically significant

qRT-PCR results demonstrated that ALP mRNA and miR-29a-3p up-regulated on day 5, peaked on day 10, and returned to normal on day 20, while Runx2 mRNA increased on day 5, peaked on day 15, and returned to normal on day 20. As we expected, the expressions of these genes were significantly decreased in HF group compared to the normal group, especially on day 10 (Fig. 1b). Taken together, we demonstrated that hyperlipidemia hindered osseointegration was correlated with deregulated miR-29a-3p expression.

### MiR-29a-3p overexpression enforced osseointegration and miR-29a-3p inhibitor repressed osseointegration in vivo

**Fig. 2:**
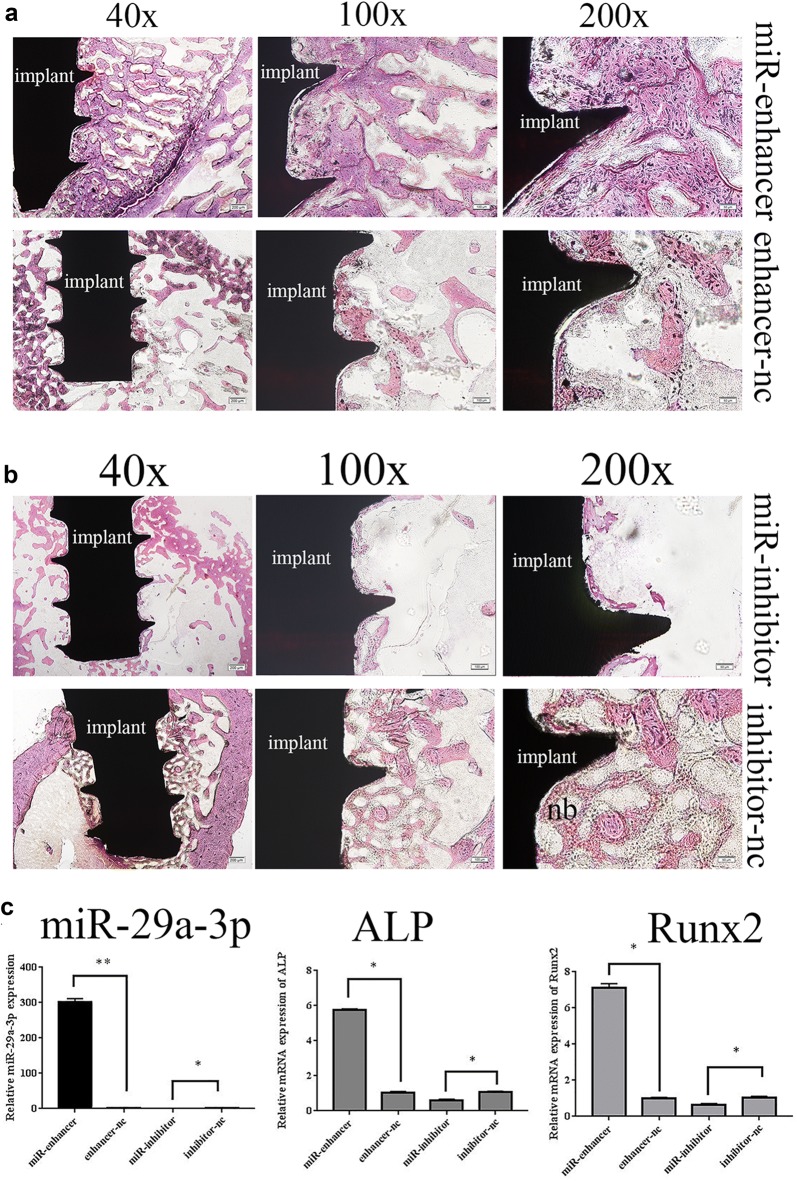
MiR-29a-3p overexpression facilitated osseointegration and miR-29a-3p inhibition suppresses osseointegration in vivo. Hard tissue slices of implant periphery miR-29a-3p overexpressed bone and overexpression-nc bone (**a**) and miR-29a-3p inhibited bone and inhibitor-nc bone (**b**). Relative expression levels of miR-29a-3p, ALP mRNA and Runx2 mRNA in miR-29a-3p-enhancer, miR-29a-3p-inhibitor or enhancer/inhibitor-nc bone tissues (**c**). Results are represented as mean ± SD. *P < 0.05, **P < 0.01

To further explore the function of miR-29a-3p on osseointegration, miR-29a-3p gain- and reduce-of-function assays were performed in hyperlipidemia models. Implants were surrounded by abundant newly formed woven bone, and bone mass, new bone volume was increased on miR-29a-3p overexpressed group, while a bare amount of bone tissue was observed in the implant-bone interface of miR-29a-3p inhibited group (Fig. 2a, b). qRT-PCR results demonstrated that miR-29a-3p, ALP and Runx2 mRNAs were dramatically up-regulated in miR-29a-3p-enhancer treated group (miR29a-3p 34-folds, ALP 5.7-folds, Runx2 7.6-folds) compared to the control group. Conversely, these genes were significantly declined when transfected with miR-29a-3p-inhibitor (miR-29a-3p 0.36-folds, ALP 0.43-folds, Runx2 0.62-folds) compared to the inhibitor-nc group (Fig. 2c). Collectively, these results suggested that hyperlipidemia impaired osseointegration and reduced miR-29a-3p expression, while with the expression of miR-29a-3p up-regulated, osseointegration was promoted.

### Hyperlipidemia weakened BMSCs’ osteogenesis in vitro

**Fig. 3:**
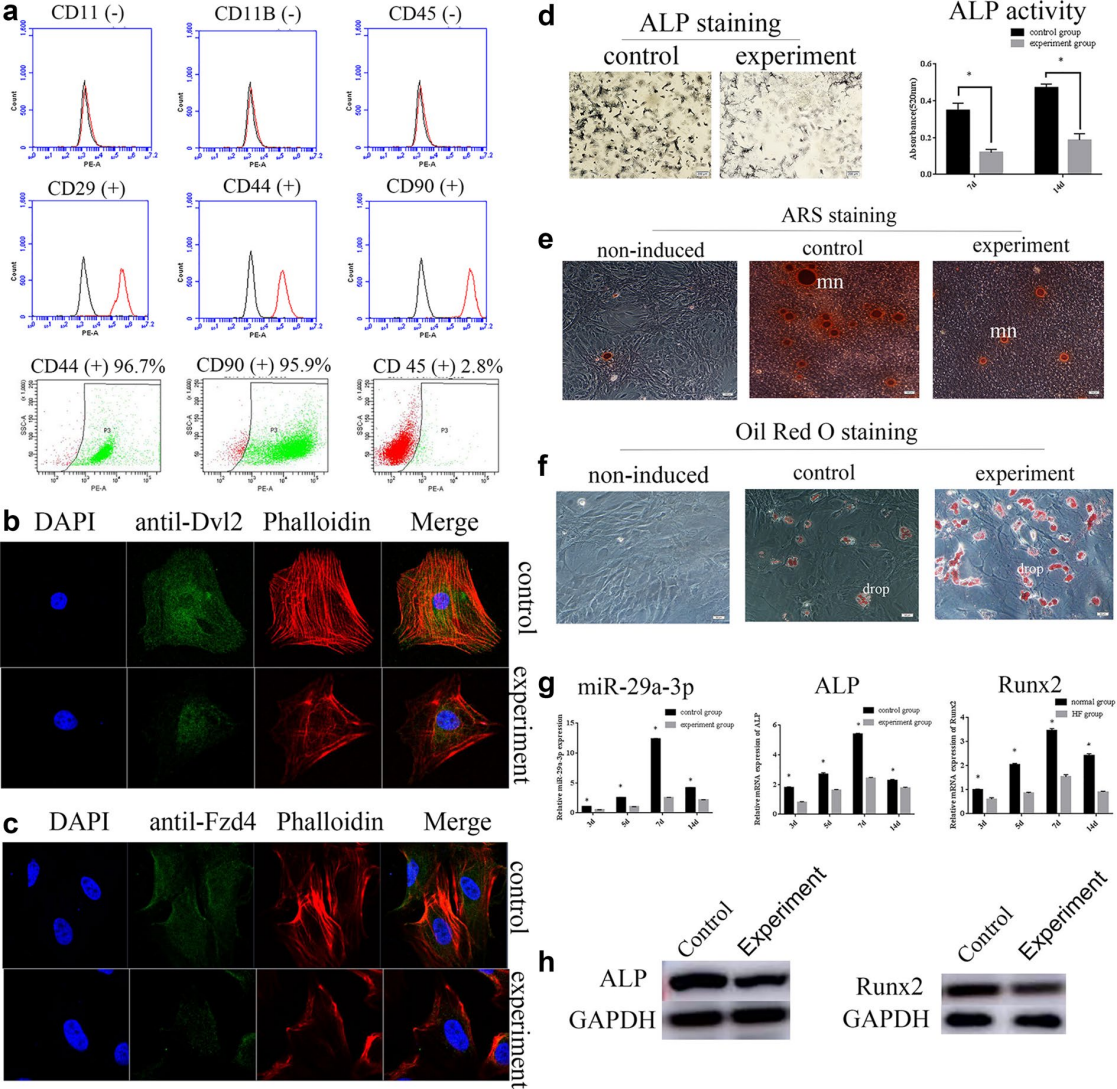
MiR-29a-3p expression was decreased in experiment group correlated with compromised osteogenic differentiation. **a** FACS was performed to identify BMSCs and assess the purity of BMSCs. Immunofluorescence was carried out to show the cytoskeletal structures and the introcellular location of ALP proteins (**b**), and Runx2 proteins (**c**). **d** ALP staining and ALP activity assays were applied to detect osteogenic differentiation. **e** ARS staining was used to determine the mineralization of osteoblasts, mn: mineralized nodule. **f** Oil red O staining was conducted to analyze the adipogenesis of osteoblasts, drop: lipid drop. **g** Relative expression levels of miR-29a-3p, ALP mRNA and Runx2 mRNA in control and experiment groups. **h** Western blotting was used to detect ALP and Runx2 proteins in control and experiment groups. Results are represented as mean ± SD of three independent experiments. *P < 0.05

Given the adverse effect of hyperlipidemia on osseointegration in rats, next we investigated the functional role of hyperlipidemia in vitro. To assess the purity of rat BMSCs, we used FACS to detect the markers of BMSCs. Our results showed that rat BMSCs expressed CD44, CD90 and CD29 (+, positive rate > 95%), CD11, CD11b and CD45 (−, positive rate < 5%), indicating that BMSCs were of high purity (Fig. 3a).

Cytoskeletons, related to bone matrix deposit, are vital for osteogenesis [41]. Immunofluorescence staining was exerted to show the cytoskeletal structure and the locations of ALP and Runx2 proteins in cells. The cytoskeletal structure of osteoblast in control group tends to be dense and fine ordered, while the experiment group gained sparse and disordered cytoskeletal structure. Moreover, ALP and Runx2 proteins were widely distributed in the cytoplasm and their expressions were significantly weakened in experiment group compared to the control group (Fig. 3b, c).

BMSCs’ osteogenic differentiation is correlated with the increased expression of intracellular ALP. To investigate the biological and functional roles of hyperlipidemia in osteogenic differentiation in vitro, ALP staining and ALP activity assays were conducted in BMSCs 7 days after induction. ALP staining showed that the activity of ALP on day 7 was significantly decreased in experiment group in comparison to control group (Fig. 3d). Consistently, ALP activity assays showed that ALP activity in experiment group was considerably lower than the control group both on day 7 and 14 (Fig. 3d).

Osteogenesis was accompanied by mineralization. ARS staining was performed to assess the mineralization function of osteoblasts. The density, volume and quantity of mineralized nodules were reduced in the experiment group in comparison to the control group (Fig. 3e), while little mineralized nodules were observed in the non-induced group. These results indicated that there was a lower level of mineralization of osteoblasts in the experiment group.

Additionally, high standard adipogenesis is correlated with low standard osteogenesis [1–3]. Oil red O staining was used to examine adipogenesis of non-induced, control and experiment groups. A large amount of lipid drops were observed in the experiment group, while only a few lipid drops existed in the control group and barely no lipid drops were observed in the non-induced group (Fig. 3f). These results demonstrated that hyperlipidemia facilitates adipogenesis, which further confirmed that hyperlipidemia impaired osteogenic differentiation of BMSCs.

### Hyperlipidemia reduced the expression of miR-29a-3p during osteogenesis in intro

qRT-PCR showed miR-29a-3p and ALP, Runx2 mRNAs increased on day 3, summited on day 7, and then decreased to normal on day 14. Importantly, the gene expressions were lower in the experiment group than in the control group, especially on day 7 (miR-29a-3p 0.2-fold, ALP 0.48-folds, Runx2 0.35-folds) (Fig. 3g). Similarly, Western blotting showed the reduction of ALP and Runx2 proteins in the experiment group in comparison to the control group (Fig. 3h). These results indicated that miR-29a-3p was suppressed by hyperlipidemia during BMSC osteogenesis.

### Overexpressed miR-29a-3p increased osteogenesis while downexpressed miR-29a-3p decreased osteogenesis

**Fig. 4:**
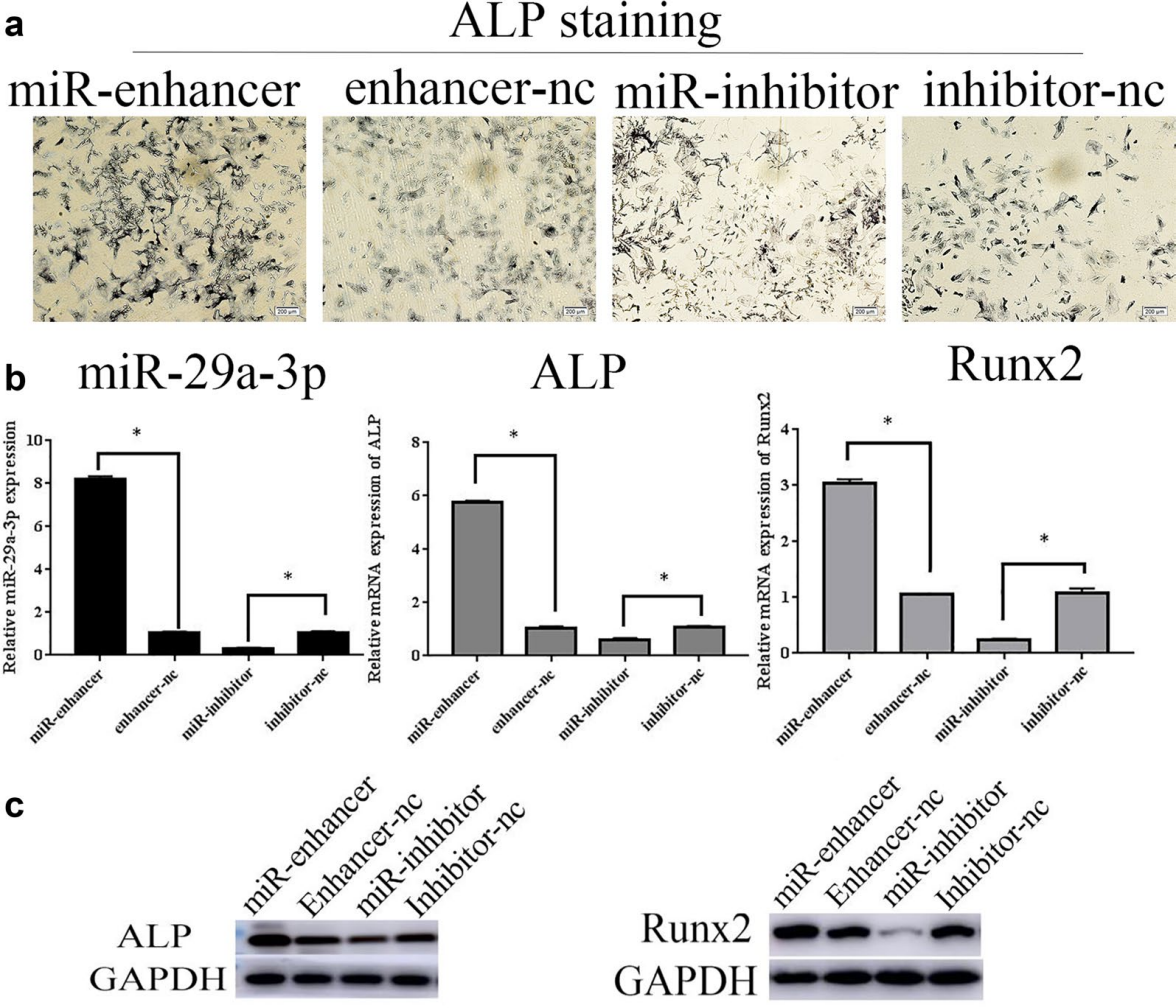
Overexpressed miR-29a-3p forced osteogenic differentiation and miR-29a-3p inhibitor reduced differentiation in vitro. BMSCs were transfected with miR-29a-3p enhancer, enhancer-nc, miR-29a-3p inhibitor, or inhibitor-nc, followed by the measurement of osteogenic differentiation by ALP staining (**a**), miR-29a-3p, ALP mRNA and Runx2 mRNA (**b**), and ALP and Runx2 proteins (**c**). Results are represented as mean ± SD. *P < 0.05

ALP staining delineated that ALP activity was greatly increased in cells transfected with miR-29a-3p-enhancer and suppressed by miR-29a-3p inhibitor (Fig. 4a). qRT-PCR showed the up-regulation of miR-29a-3p, ALP and Runx2 mRNA (miR-29a-3p 8.1-folds, ALP 5.8-folds, Runx2 3.0-folds) of BMSCs transfected with miR-29a-3p-enhancer in comparison to the enhancer-nc group, while miR-29a-3p-inhibitor down-regulated these genes (miR-29a-3p 0.31-fold, ALP 0.057-fold, Runx2 0.22-folds) compared to the inhibitor-nc group. As we expect, western blotting also showed that ALP, Runx2 proteins were up-regulated in the miR-enhancer group and down-regulated in the miR-inhibitor group (Fig. 4b, c). Together, overexpression of miR-29a-3p promoted osteogenesis and deregulated miR-29a-3p suppressed osteogenesis in vitro.

### MiR-29a-3p directly suppressed Dvl2 and Fzd4 in osteogenesis

**Fig. 5:**
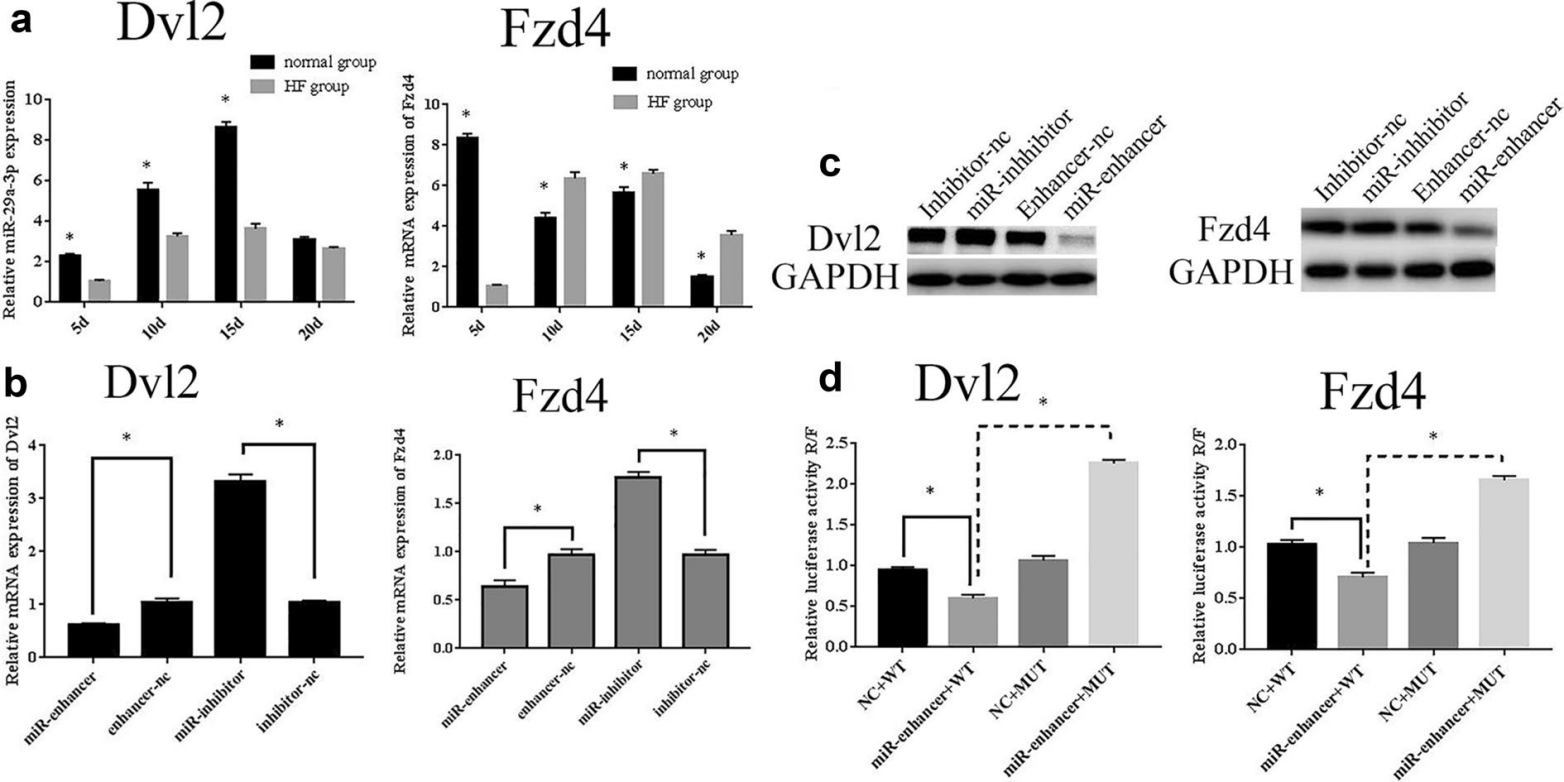
miR-29a-3p directly suppressed Dvl2 and Fzd4 expression by binding to their 3′-UTR. **a** mRNA levels of Dvl2 and Fzd4 in peri-implant bone tissue in hyperlipidemia and normal rat models. The mRNA levels of Dvl2 and Fzd4 (**b**) and protein levels of Dvl2 and Fzd4 (**c**) in BMSCs transfected with miR-29a-3p enhancer, enhancer-nc, miR-29a-3p inhibitor, or inhibitor-nc. **d** Luciferase reporters assay was performed in 293T cells after transfected with miR-29a-3p-WT enhancer reporter, miR29a-3p-MUT enhancer reporter or miR-WT/MUT NC. Results are represented as mean ± SD. *P < 0.05

By using different databases (miRDB, Target Scan, MicroRNA.org, Microcosm Targets), we predicted that miR-29a-3p could directly suppress Dvl2 and Fzd4. Firstly, qRT-PCR demonstrated that Dvl2 mRNA started to ascend on day 5, submitted on day 15, and returned to normal on day 20 both in HF and normal rats, and Dvl2 mRNA was slightly suppressed in the HF group compared to the normal group on each time point (Fig. 5a). Fzd4 mRNA in the normal group was dramatically declined from day 5 to day 10, then upregulated until day 15, and returned to normal on day 20. Fzd4 mRNA on HF group started to increase on day 5, peaked on day 15, and returned to normal on day 20 (Fig. 5a). Next, to verify whether miR-29a-3p could directly bind to Dvl2 and Fzd4, qRT-PCR, western blotting were conducted in BMSCs and dual luciferase reporter gene assay was conducted in 293T cells. qRT-PCR and Western blotting demonstrated that overexpressed miR-29a-3p repressed the Dvl2 and Fzd4 mRNA and protein expressions, while miR-29a-3p-inhibitor enhanced their expressions (Fig. 5b, c). Furthermore, dual luciferase reporter assays revealed that miR-29a-3p-enhancer significantly decreased the luciferase activity of wild-type 3′-UTR compared to the miR-NC group. Strikingly, this inhibition was revoked when the miRNA response element was mutated (Fig. 5d). All these data suggested that miR-29a-3p directly interacted with Dvl2 and Fzd4 3′-UTR promoter region to regulate its expression.

## Discussion

Previous studies have discovered that hyperlipidemia was closely associated with bone and bone disease and a variety of miRNAs have been demonstrated to affect osteogenesis through modulating osteogenic differentiation, bone matrix deposition, cytoskeleton formation and osteoclast metabolism [1–3, 20–24]. MiR-29a-3p was previously suggested to facilitate osteogenic differentiation through activating Wnt/β-catenin signal pathway [27]. However, further explorations on the biological significance of hyperlipidemia in osseointegration, as well as the underlying mechanisms of miR-29a-3p in regulating osseointegration and osseointegration are required.

Keuroghlian et al. [3] demonstrated that hyperlipidemia significantly increased implant loss and decreased the formation and strength of the bone-to-implant interface in the mouse femur, which were supported by such observations that increased implant failure, decreased osseointegration, and poor mechanical strength were associated with untreated hyperlipidemia in patients. In addition, Pirih et al. delineated that hyperlipidemia induces secondary hyperparathyroidism and impairs bone regeneration and mechanical strength [2]. Here, we found that osseointegration was significantly hindered by hyperlipidemia in a rat model, further confirmed previous reports in human and mice.

A great many of studies highlighted that miR-29a-3p modulate BMSCs proliferation and differentiation. MiR-29a-3p facilitated transplantation of BMSCs to alleviate pelvic floor dysfunction by repressing elastin [4]. In addition, miR-29a-3p was significantly deregulated in the serum of patients with low bone mass compared with controls [42]. Moreover, miR-29a-3p expression increased during osteoblasts mature process correlated with activated Wnt signaling [27]. In the current study, we found that decreased miR-29a-3p in hyperlipidemia rats correlated with impaired osseointegration. Moreover, subsequent gain- and reduce-of-function experiments demonstrated that miR-29a-3p overexpression enhanced osseointegration and osteoblastic differentiation, while inhibition of miR-29a-3p obtained opposite result. Our previous study demonstrated that miR-29c-3p promoted osteogenic differentiation of BMSCs by directly suppressing Dvl2 in the Wnt/β-catenin signal pathway-related [40], which played critical roles in bone formation, reconstruction and maintenance [43–46].

Wnt/β-catenin pathway enhanced bone formation by up-regulating osteogenic differentiation via promoting the expression of osteogenesis related genes, such as Runx2, ALP and OPN (osteoprotegerin) [12–15, 47]. In addition, Wnt/β-catenin pathway enhanced the activity through binding to the 7-transmembrane frizzled (Fzd) receptor and low-density lipoprotein 5 and 6 (LRP5/6) co-receptors, which in turn recruited the most proximal signaling intermediate, Dvl, to the plasma membrane [43, 48]. Based on this, we further tested whether miR-29a-3p could regulate osseointegration through suppressing predicted targets Dvl2 and Fzd4. Indeed, we confirmed that miR-29a-3p could suppress Dvl2 and Fzd4 through directly binding their 3′-UTRs. To our knowledge, this was the first experiment to verify the direct inhibition of miR-29a-3p to Dvl2 and Fzd4 during osseointegration.

Dvl2 played a critical role in the Wnt signal transduction pathways [40, 49, 50]. The activity of Dvl2 was up-regulated upon its phosphorylation [51], and down-regulated by ubiquitination [52]. We found in our previous study that hyperlipidemia reduced osseointegration in rats via suppressing the expression of phosphor-Dvl2 as well as promoting expression of ubiquitinated-Dvl2. In this study, we showed that there was a significant reduction of Dvl2 during osseointegration in normal rats in comparison to hyperlipidemia rats. What is more, the expression of Fzd4 fluctuated in the normal and HF groups in vivo, and Fzd4 expression was significantly higher at the middle stage of osseointegration in hyperlipidemia rats than in normal rats. Several studies elucidated that Fzd4 plays a role in osteogenesis. For example, Long et al. demonstrated that miR-139-5p inhibited BMSCs’ osteogenesis via directly targeting Fzd4 and β-catenin [53]. In addition, Fzd4 was engaged in Wnt5a mediated orthodontic force induced bone remodeling via activating Wnt/β-catenin signaling pathway [54]. Furthermore, Dvl2 could recruit β-Arrestin 2, ubiquitous cellular scaffolding proteins, to mediate Wnt5a-stimulated endocytosis of Fzd4 [48], further indicating the significant importance of the interaction between Dvl2 and Fzd4 during the activation of Wnt signal pathway.

Nevertheless, though the present study has demonstrated that Dvl2 and Fzd4 were the direct target of miR-29a-3p, the underlying mechanisms of miR-29a-3p targeting Dvl2 and Fzd4, and the interactions between Dvl2 and Fzd4 remained unclear. Further studies are needed to elucidate these molecular mechanisms.

## Conclusion

In conclusion, our results showed the adverse effect of hyperlipidemia on osseointegration in rats. Moreover, we demonstrated that miR-29a-3p enforced osseointegration by targeting Dvl2 and Fzd4 in the Wnt/β-catenin pathway, and this enforcement could be impaired by hyperlipidemia.

## Abbreviations

BMSCs: bone marrow mesenchymal stem cells
miRNAs: microRNAs
HF: high-fat
HFD: high-fat diet
miR-29a-3p: microRNA-29a-3p
ALP: alkaline phosphatase
Runx2: runt-related transcription factor 2
Dvl2: dishevelled 2
Fzd4: frizzled 4
qRT-PCR: real-time qantitative PCR
PVDF: polyvinylidene difluoride
FACS: fluorescence-activated cell sorting
